# Induction of Osteogenic Differentiation in Human Mesenchymal Stem Cells by Crosstalk with Osteoblasts

**DOI:** 10.1089/biores.2015.0002

**Published:** 2015-01-01

**Authors:** Martina Glueck, Oliver Gardner, Ewa Czekanska, Mauro Alini, Martin J. Stoddart, Gian M. Salzmann, Hagen Schmal

**Affiliations:** ^1^ AO Research Institute Davos, Davos Platz, Switzerland.; ^2^ Department of Orthopaedic and Trauma Surgery, University Medical Center, Albert-Ludwigs University Freiburg, Germany.; ^3^ Cardiff University, Cardiff, Wales, United Kingdom.

**Keywords:** mesenchymal stem cell, osteoblast, co-culture, osteogenic differentiation, bone formation

## Abstract

Natural bone healing following fractures is initiated by osteoblasts (OBs) and mesenchymal stem cells (MSCs), a cell combination with possible potential in tissue engineering techniques for bony defects. The aim of the study was to investigate MSC/OB-crosstalk, in order to determine optimal cell culture conditions for osteogenic differentiation. Human OBs and MSCs interactions were investigated in an *in vitro* trans-well co-culture study over a time period of 28 days. Calcification was determined by optical density (OD) at 450 nm and Alizarin red staining. Messenger RNA expression was assessed by quantitative PCR. Osteogenic medium containing 1% fetal bovine serum resulted in superior levels of calcification in MSCs in co-culture with OBs compared to 2% or 5% fetal bovine serum (*p*<0.05). Comparing MSCs and OBs alone with the MSC/OB co-culture, calcification, as measured by OD 450 nm, increased over time in all groups. The highest values were recorded in the co-culture (*p*<0.05). Osteogenic differentiation potential showed significant interindividual differences. In order to predict differentiation potential, OD 450 nm measurements and mRNA expression of alkaline phosphatase were correlated with the population doubling rate during the expansion period. For OBs and MSCs, statistically significant associations of proliferation and differentiation potential were found (*p*<0.001). The addition of transforming growth factor beta resulted in up-regulation of collagen type I and Sp7 mRNA, and down-regulation of alkaline phosphatase mRNA. The results suggest the idea of soluble paracrine factors being secreted by OBs to induce osteogenic differentiation of MSCs.

## Introduction

Isolated bone or osteochondral defects in joints are seen following osteochondritis dissecans or traumatic injuries and are associated with a significant risk for development of consecutive osteoarthritis or non-unions. Therefore, these lesions need to be addressed by surgical repair.^1^ The multipotent nature of mesenchymal stem cells (MSCs),^2^ makes them an ideal candidate for cell therapy for musculoskeletal applications including bone regeneration. This is supported by their immune-suppressive regulatory properties,^3^ a characteristic that might be useful in cases with activation of inflammatory responses following acute impacts or fracture repair. Another theoretical advantage is the described higher tolerance of hypoxia compared with fully differentiated osteoblasts, allowing more time for the formation of a vascular network to supply the bone component.^4^ Knowledge about MSC differentiation in the vicinity of differentiated osteoblasts will also help to understand how natural repair of bony defects might function. Differentiation of MSCs may be controlled by different stimuli in cell culture media.^2^ Transforming growth factor beta (TGFβ) is a key component regulating osteochondral differentiation and is prevalent during fracture repair. Dexamethasone/β-glycerolphosphate have been shown to be required for osteogenic differentiation.^5^ A recently described opportunity for induction of an osteogenic phenotype^6,7^ is the co-culture with differentiated cells, a phenomenon that possibly plays an important role for the formation of the bone marrow niche. Here both cell–cell contacts and paracrine secretion mechanisms appear to play a significant role.^6–8^

The intention of this project was the definition of conditions that may support bone formation in a co-culture of MSC and human osteoblasts (OBs). The influence of serum concentrations, cell functionality, and the presence of TGFβ were all investigated.

## Material and Methods

### Isolation of OBs and MSCs

Femoral heads were obtained during hip arthroplasty operations following femoral neck fractures. The material used was obtained with informed consent from patients in accordance with the University of Freiburg Medical Center Ethics Committee (Tissue Bank for Research in the Field of Tissue Engineering project [GTE-2002]). The degrees of osteoarthritis were evaluated on X-rays using Croft's modification of the Kellgren and Lawrence grading system. Cells from patients with advanced osteoarthritis (≥grade 3) were not used for the experiments. Within 8 hours post–surgery osteoblasts [OBs] and mesenchymal stem cells [MSCs] were separated from one femoral head as previously described.^9^

Human primary osteoblasts were isolated from cancellous bone of the femoral heads by cell outgrowth from small pieces.^10^ MSCs were extracted from the bone marrow of cancellous bone of the same femoral heads.^5^ Briefly, for MSC isolation small pieces of cancellous bone were transferred in a conical tube with medium and vortexed. This step was repeated four times. Afterwards, mononuclear cells were separated by a Ficoll-Paque gradient (Pharmacia, Piscataway, NJ), washed and seeded in flasks. For OB isolation, cells were additionally released from bone pieces by a short trypsin/EDTA digestion and seeded in flasks without further enrichment. Cells were aliquoted and stored frozen in liquid nitrogen until experiments were performed. The time from rethawing until the beginning of the experiment is referred to as the expansion period and differed among donors between 1 and 3 weeks due to differences in proliferation rates. MSCs were thawed 7–10 days later than the OBs due do the more rapid proliferation rate. During the expansion period, MSCs were cultured in Alpha Minimum Essential Medium (Gibco, Carlsbad, CA), 10% fetal bovine serum (FBS) (MSC qualified, Gibco), 5 ng/mL FGF-2 (Invitrogen, Karlsruhe, Germany), 1% penicillin/streptomycin. OBs were cultured in Dulbecco's modified Eagle's medium (DMEM)/ low glucose (1g/L, Gibco), 10% FBS (heat inactivated, Gibco) and 1% penicillin/streptomycin.

### Experimental setup

MSCs in the bottom and OBs on top were separated in a trans-well culture with 0.4 μm inserts. As a basal culture medium DMEM/low glucose (1 g/L, Gibco) without phenol-red, supplemented with ascorbic acid 50 μg/mL, β-glycerolphosphate 5 mM, dexamethasone 1×10^–7^ M, nonessential amino acids 1%, ITS 1%, HEPES buffer 20 mM, and penicillin/streptomycin 1% was used. Half media changes were performed three times per week. The initial seeding density was 20,000 cells/cm^2^. The cells from the different donors were combined as indicated in Table 1 and were used at a maximum passage of 4. The total time of co-culture was 28 days.

**Table 1.** **Cell Combinations Used in Each Run of the Experiment**

	Experiment run 1	Experiment run 2	Experiment run 3
MSC	♀, age 73 years	♀, age 83 years	♂, age 76 years
OB	♂, age 76 years	♂, age 72 years	♀, age 73 years

MSC, mesenchymal stem cells; OB, human osteoblasts.

The effect of adding FBS to the basal culture medium in increasing concentrations of 1%, 2%, and 5% was tested. In order to examine the effect of TGFβ, the medium was additionally supplemented with 2 ng/mL TGFβ-1 (Invitrogen) during the whole culture period.

### Quantitation of osteogenic differentiation

Calcification was assessed using two independent methods. At first, a spectroscopic analysis was done measuring the absorbance (optical density) at 450 nm to allow for nondestructive online measurement. Calcium and phosphate deposition marking the beginning of the bone formation process, in combination with the increasing cell density, leading to a higher absorbance.^11^ Measurements were taken at day 7, 14, 21, and 28 from the cells within the wells. Secondly, the more specific Alizarin red staining (ARS) of the cells within the wells at day 28 and a subsequent quantitative assay were done. Alizarin red working solution (Sigma-Aldrich, St. Louis, MO) was prepared to a concentration of 40 mM and a pH of 4.1–4.3. After 28 days of culture the samples were fixed with 4% formaldehyde, washed, incubated with the dye for 30 min on a rotating plate, and then washed with double distilled water. For quantification, the stained monolayer was dissolved in 10% acidic acid. The suspension was heated to 85°C for 10 min and centrifuged at 20,000 *g* for 15 min after cooling on ice. The supernatant was collected and pH was adjusted to 4.2 with ammonium hydroxide. For quantification, the samples' absorption at an optical density (OD) of 405 nm was plotted against a standard.

The population doubling rate (PDR) was used to compare proliferation capacity among cells of different donors. PDR was calculated for the pre-culture expansion time from thawing until seeding the cells using the formula: 3.32×(LOG[cell count last day of preculture]−LOG[cell count first day of preculture]) divided by the day number of preculture period.

### Quantitative PCR

Gene up-regulation by day 28 compared to day 0 (seeding day) was analyzed by RT-PCR using 18S rRNA as a housekeeping gene. At day 0 and 28 approximately 1×10^6^ OBs and MSCs were taken for separate RNA isolation. The cell pellets were resuspended in 1 mL TRI Reagent (MRC Global Inc., Houston, TX) and 5 μL of polyacryl carrier (MRC Global Inc.) was added. At day 28 the top cells growing in the co-culture insert were washed, dissolved in 300 μL TRI Reagent and 5 μL of polyacryl carrier was added. Alteration of gene expression was measured on the MSCs co-cultured with OBs (MSC_OB). MSCs co-cultured with MSCs (MSC_MSC) were used as negative control whereas OBs co-cultured with OBs (OB_OB) served as positive control. RNA was isolated by phase separation with 1-Bromo-3-chloropropane (Sigma-Aldrich) and several subsequent washing steps. For reverse transcription, the TaqMan Reverse Transcription Reagent kit (Invitrogen) with 1 μg total RNA was used with random oligomers as per manufactures instructions. For PCR, TaqMan Gene Expression Master Mix (Applied Biosystems, Carlsbad, CA) and human primers and probes (all from Applied Biosystems) with the sequences shown in Table 2 were used.

**Table 2.** **Overview of Primers and Probes Used**

Gene	Primer forward (5′-3′)	Primer reverse (5′-3′)	Probe (5′FAM/3′TAMRA)
18S rRNA	rRNA endogenous control, VIC/TAMRA, 4310893E-1105050, X03205.1		
ALP	Assay on demand, FAM, Hs00758162_m1, PN4351370		
COL1A1	CCC TGG AAA GAA TGG AGA TGA T	ACT GAA ACC TCT GTG TCC CTT CA	CGGGCAATCCTCGAGCACCCT
Sp7	CCT GCT TGA GGA GGA AGT TCA	GGC TAG AGC CAC CAA ATT TGC	TCC CCT GGC CAT GCT GAC GG

ALP, alkaline phosphatase; COL1A1, collagen type 1.

### Statistical analysis

All values of one experiment were expressed as mean±standard deviation and each experiment was repeated three times. Individual group medians were compared with the Mann-Whitney U rank sum test. For multiple comparisons, post hoc statistic (Kruskal-Wallis H-test) was used to analyze statistical significances between the grouped values. Correlations were determined by calculating the Spearmen coefficient (ρ); the coefficient of determination (R^2^) was calculated by means of a regression analysis. Statistical significance was defined when *p*<0.05.

## Results

### Influence of serum content on osteogenic differentiation

**FIG. 1.**
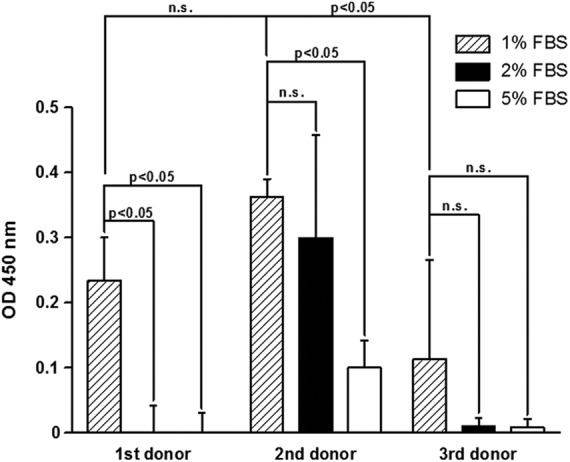
The figure shows the effect of serum concentration in a trans-well co-culture for mesenchymal stem cells (MSCs) in the bottom with osteoblasts (OBs) loaded on top. A spectroscopic analysis measuring the absorbance at 450 nm was used in order to quantitate the calcification. After 28 days for all three donors, the highest degree of calcium deposition was found using 1% fetal bovine serum (FBS) added to the culture medium. OD, optical density, n.s., not significant.

The effect of serum concentration in a trans-well co-culture for MSCs in the bottom (b) with OBs loaded on top (t) was examined. A spectroscopic analysis measuring the absorbance at 450 nm was used in order to monitor the calcification. After 28 days for all three donors, the highest degree of calcium deposition was found using 1% FBS (heat inactivated, 0.36±0.06, donor 2) added to the culture medium compared to 2% (0.30±0.16, donor 2) and 5% (0.10±0.04, donor 2). Statistical significances were found as indicated in Figure 1. Although the effect of FBS concentration was reproducible using cells from different donors, there also were remarkable differences between the donors. Optical density measured for the cultures with 1% FBS reached the highest absorbance in donor 2 followed by donor 1 (0.23±0.06, p=0.248) and donor 3 (0.11±0.15, p=0.0495). For the 2% concentration, the measurements reached statistical significance for all donors.

**FIG. 2.**
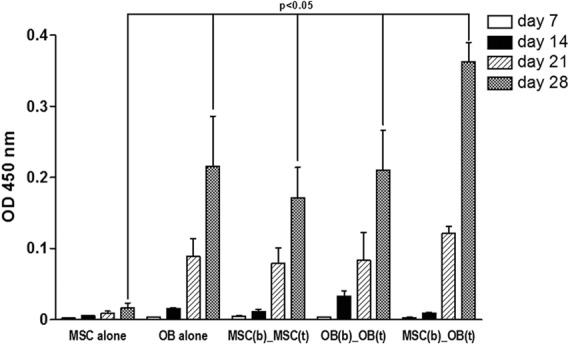
An increasing OD at 450 nm over the 4-week culture period could be observed in all groups [Kruskal-Wallis H-test *p*<0.025, 1% FBS) (MSCs alone as negative control; OBs alone as positive control, co-culture of MSCs in the bottom (b) with MSCs loaded on top (t), MSC(b)_MSC(t); co-culture of OBs in the bottom (b) with OBs loaded on top (t), OB(b)_OB(t); co-culture of MSCs in the bottom (b) with OBs loaded on top (t), MSC(b)_OB (t)]. MSCs in co-culture with OBs showed the highest absolute value of extinction at 450 nm by day 28 compared with all other groups (*p*=0.049).

An increasing OD at 450 nm over the 4 weeks culture period could be observed in all groups [MSCs alone as negative control, OBs alone as positive control, co-culture of MSCs in the bottom (b) with MSCs loaded on top (t), MSC(b)_MSC(t); co-culture of OBs in the bottom (b) with OBs loaded on top (t), OB(b)_OB(t); co-culture of MSCs in the bottom (b) with OBs loaded on top (t), MSC(b)_OB (t)] as shown in Fig. 2 (representative experiment, Kruskal-Wallis H-Test, *p*<0.025 for all groups).

MSCs in co-culture with OBs showed the highest absolute value of extinction at 450 nm by day 28 compared with all other groups (0.36±0.03, p=0.049). This maximum of OD at 450 nm was followed by OBs alone (0.22±0.07); OB(b)_OB(t) (0.21±0.06); MSC(b)_MSC(t) (0.17±0.04); and MSCs alone (0.02±0.01). Data show the remarkable effect of the co-culture by itself on the osteogenic differentiation of MSCs, comparing the indicated values for the extinction by MSCs alone and MSC(b)_MSC(t). At day 14 OD at 450 nm was still higher in the co-culture of OBs compared to MSCs, but after this time point extinction values equaled each other. This observation was independent of serum concentration and donor.

**FIG. 3.**
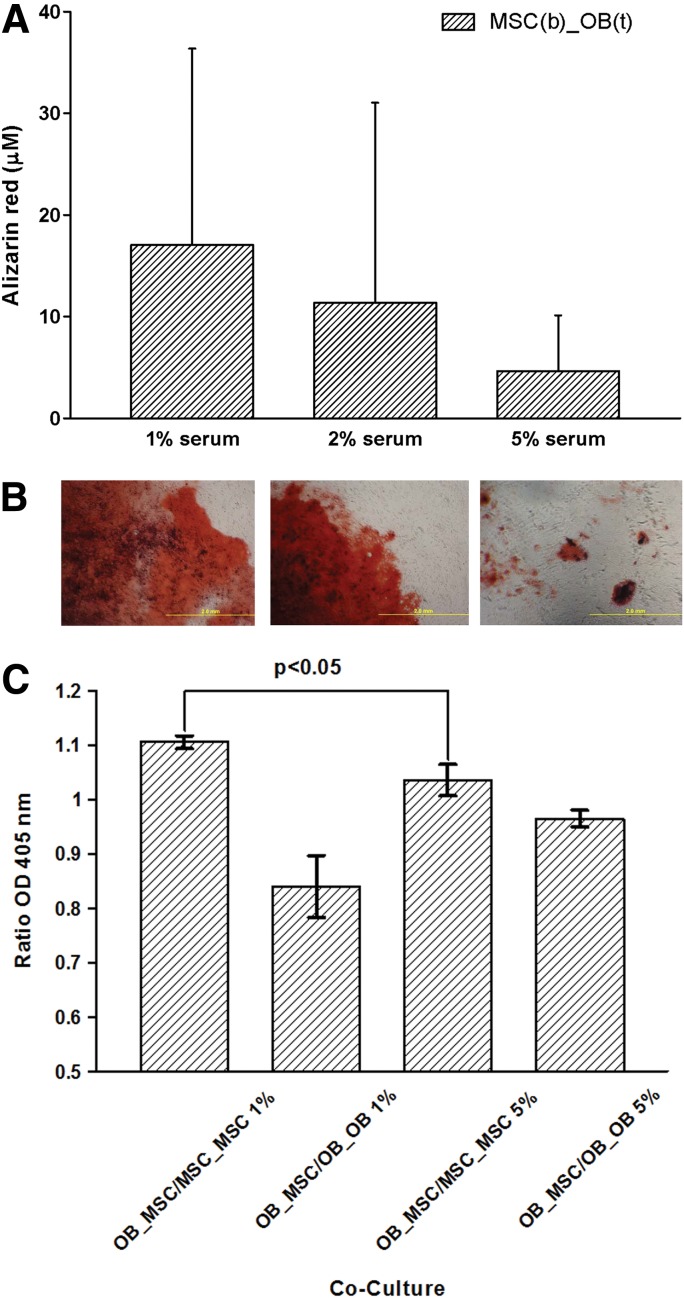
Microscopy at day 28 for Alizarin red staining **(A)** of MSCs in the bottom wells of a co-culture with OBs on top showing increasing calcification with decreasing serum concentration. This was confirmed by the subsequent quantitative assay at day 28 **(B)**. A new calculation based on individual ratios was added in order to eliminate the variations between the different donors. Correlating the OD 450 data, this resulted in a statistically significant higher intensity of Alizarin red staining of the co-culture (OB_MSC) using 1% serum compared to 5% **(C)**.

The data of OD 450 nm measurements correlates with ARS of cultures within the culture well (Fig. 3A, B). This was confirmed by the subsequent quantitative assay at day 28 showing the highest absorbance and amount of Alizarin red dye for the co-culture containing 1% FBS (17.0±19.3 μM) compared with 2% (11.4±19.4 μM) and 5% FBS (4.6±5.5 μM) (Fig. 3A). Data only mark a trend, as no statistically significant differences between the groups were observed. Interestingly, there was a difference between the MSC/OB co-culture and the MSC control, because spontaneous calcification of MSC alone was higher using 2% serum concentrations (0.00±0.00 μM vs. 2.35±4.10 μM). This analysis resulted in very high standard deviations caused by marked interindividual differences. Therefore, we carried out calculations based on the ratios of our investigation group (co-culture of OB and MSC [OB_MSC]) with the negative control (co-culture of MSC and MSC [MSC_MSC]) and the positive control (co-culture of OB and OB [OB_OB]) calculated for each individual. Based on this calculation, ARS was higher in the 1% group (1.1±0.01) compared with the 5% group (0.84±0.06, *p*<0.05, Fig. 3C). Furthermore, the difference between positive and negative control ratios was also statistically significant different for the 1% and the 5% group (*p*<0.05). Even the positive control of the 5% group showed statistically significant less calcification (ARS) than the positive control ratio of the 1% group.

### Influence of cell function on osteogenic differentiation

**FIG. 4.**
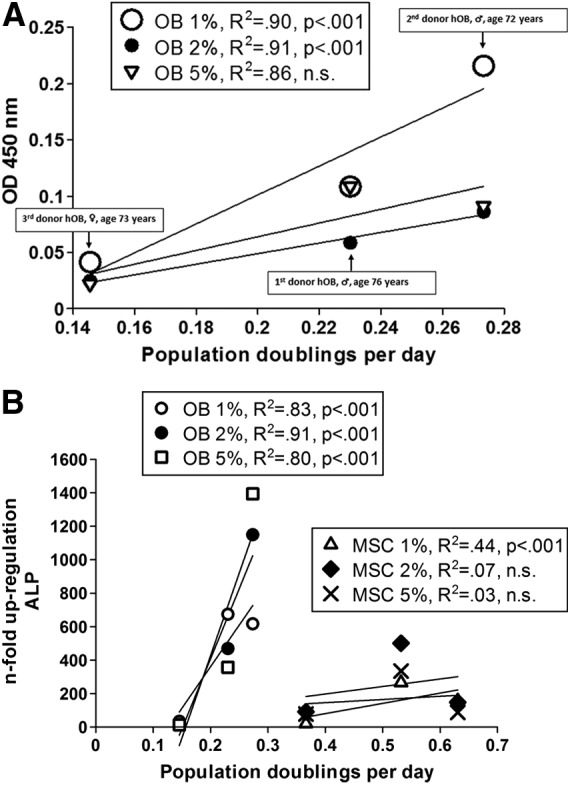
**(A)** The association of the population doubling rate during the expansion period with the OD at 450 nm is shown, demonstrating that donor-dependent proliferation determines ability for osteogenic differentiation. This could be shown for all investigated serum concentrations as indicated in the legend. hOB, human osteoblasts. **(B)** The association of the population doubling rate during the expansion period with alkaline phosphatase (ALP) mRNA expression is shown, demonstrating that donor-dependent proliferation determines ability for osteogenic differentiation. This could be shown for all investigated serum concentrations and for both cell types (MSC right and OB left) as indicated in the legend.

Although the principal effects of serum concentration on calcification were the same independent of the donors, a substantial variation of the interindividual absolute values was observed. This fact initiated the examination of a possible association of proliferation and differentiation potential. Therefore, osteogenic differentiation, represented by OD 450 nm, was correlated with the number of population doublings per day during the expansion period. Data showed that fast growing OBs with a high PDR per day showed higher values of absorbance at 450 nm after 28 days of culture, indicating better osteogenic differentiation. In Fig. 4A the OB PDR for each donor was plotted against the OD 450 nm. In the case of all three donors a strong positive correlation of PDR and the absorbance at 450 nm could be seen within the group of the same serum concentration (R^2^ for 1% FBS 0.90; R^2^ for 2% FBS 0.98; R^2^ for 5% FBS 0.74). Although the coefficients of determination indicated overall good associations, only in the groups with 1% and 2% FBS were the correlations significant (*p*<0.0001). The regression curve for the 1% FBS group had the steepest slope, indicating greater calcium deposition. This is in line with the highest value of absorption at 450 nm compared to higher serum concentrations. This also correlates with the highest amount of calcification measured by Alizarin red staining in the MSC/OB co-culture as presented in Fig. 3. Overall, fast growing OBs seem to have a higher potential to calcify compared to slowly growing OBs.

The same positive correlation between population doublings within a group of the same serum concentration could be seen for up-regulation of alkaline phosphatase (ALP) mRNA in OBs within the 28 day cell culture period measured by quantitative PCR (R^2^ for 1% FBS 0.83; R^2^ for 2% FBS 0.91; R^2^ for 5% FBS 0.80; Fig. 4B, left). The steepest slope was observed for the group with 5% FBS. The associations of all different groups reached statistical significance (*p*<0.0001). ALP up-regulation is an indicator for osteogenic differentiation, which correlates with proliferation potential. Although the coefficients of determination were much lower for MSCs, a similar association could be demonstrated for this cell type (Figure 4B, right) reaching statistical significance for the 1% FBS concentration calculating the Spearman correlation.

### Influence of TGFβ on osteogenic differentiation

**FIG. 5.**
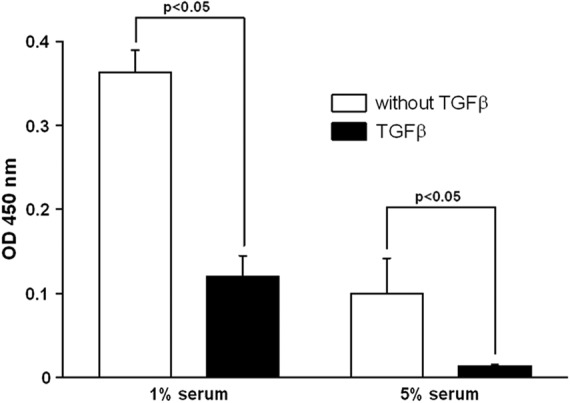
This graph shows the effect of TGFβ on calcification of MSCs in the bottom of a co-culture with OBs on top mirrored by OD 450 nm. Independent of serum concentration, a statistically significant inhibition of terminally osteogenic differentiation could be demonstrated (*p*=0.0495). TGFβ transforming growth factor beta.

The addition of TGFβ reduced the calcification in the co-culture of MSCs with OBs measured by the 450 nm absorbance (Fig. 5). This effect was not dependent on serum concentration. As seen previously, OD 450 nm was significantly higher (*p*=0.0495) after 28 days using 1% FBS (0.36±0.03) compared with 5% FBS (0.10±0.04). OD was diminished by TGFβ to 0.12±0.02 in the 1% FBS co-culture and to 0.014±0.001 in the 5% FBS co-culture (*p*=0.0495). Similarly, Alizarin red staining was reduced in the TGFβ treated cultures without reaching statistical significance (17.0±19.3 μM vs. 13.0±11.3 μM).

**FIG. 6.**
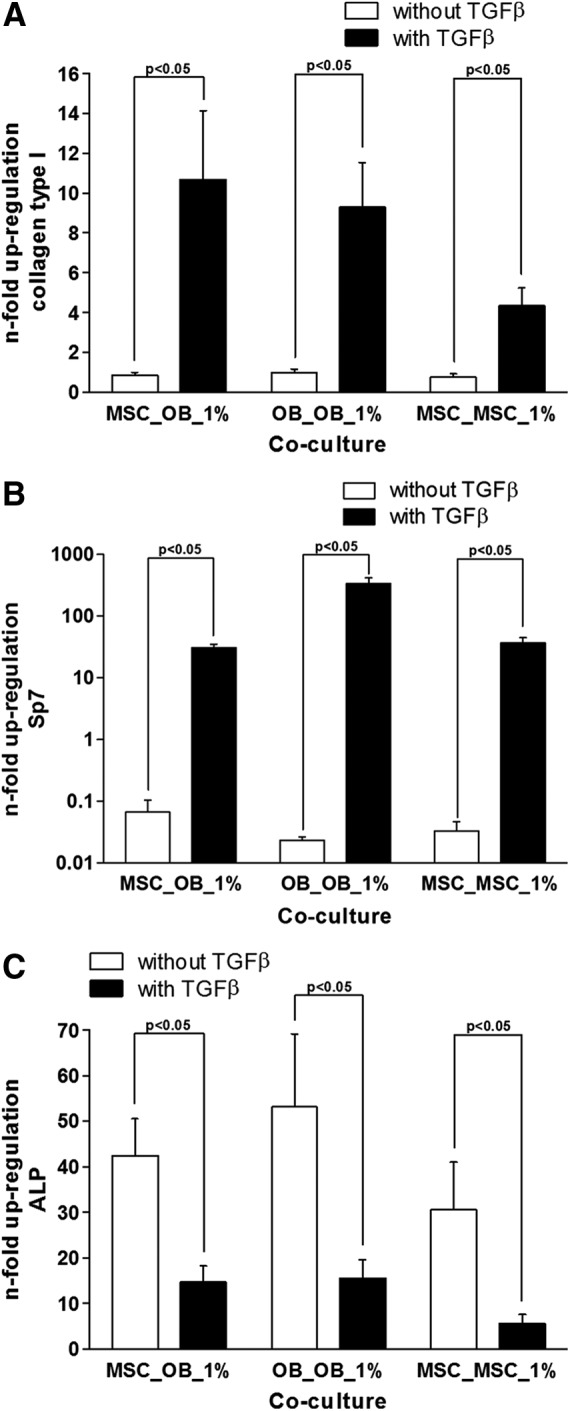
The figure shows the influence of TGFβ on the expression of the genes for collagen type 1 **(A),** the transcription factor Sp7 **(B),** and alkaline phosphatase **(C)** in co-cultures of mesenchymal stem cells (MSC) with osteoblasts (OB) (MSC_OB); OB and OB (OB_OB); and MSC and MSC (MSC_MSC) in medium containing 1% FBS. Whereas collagen type 1 (col1a) and Sp7 were up-regulated, expression of ALP was suppressed.

Real time PCR demonstrated that TGFβ caused a statistically significant up-regulation of collagen type I mRNA in all co-cultures (*n*=4 per co-culture, Fig. 6A). This effect was confirmed for both investigated cell types, MSCs and OBs. In contrast, collagen type 2 mRNA was not constitutively expressed by MSC or OB and could also not be found after the 28 days of co-culture in osteogenically differentiated MSC or other cell types. Alteration of gene expression was measured in MSCs co-cultured with OBs (MSC_OB). MSCs co-cultured with MSCs (MSC_MSC) served as the negative control, whereas OBs co-cultured with OBs (OB_OB) were regarded as the positive control. Data shown represent an experiment using 1% FBS, but the effects were constantly seen using the other investigated FBS concentrations. Addition of TGFβ caused an up-regulation of collagen type 1 mRNA (col1A1) in the MSC_OB co-culture (2.78±0.33, *p*=0.021), the OB_OB co-culture (9.29±2.25, *p*=0.021), and the MSC_MSC co-culture (4.33±0.89, *p*=0.021) compared to the non-treated samples. The lowest col1A1 expressions in the groups without TGFβ treatment were found in the MSC_OB co-culture (0.46±0.14) followed by the MSC_MSC co-culture (0.75±0.17) and the OB_OB co-culture (0.97±0.18).

It further could be demonstrated that TGFβ caused a statistically significant up-regulation of Sp7 mRNA in all co-cultures (*n*=4 per co-culture, Fig. 6B). Whereas Sp7 mRNA expression was almost completely down-regulated after 28 days cell culture compared to day 0 without TGFβ (MSC_OB 0.07±0.04, OB_OB 0.02±0.003, MSC_MSC 0.03±0.01), addition of TGFβ caused an up-regulation in the MSC_OB co-culture (30.86±3.94, *p*=0.021), the OB_OB (335.88±79.81, *p*=0.021), and the MSC_MSC co-culture (37.20±7.32, *p*=0.021). There was no statistically significant difference between the different groups without TGFβ treatment. In the groups with TGFβ there was no statistically significant difference between the MSC_OB and the MSC_MSC co-culture, but there was a statistically significant difference between both groups and the OB_OB control (*p*=0.021).

It also could demonstrated that TGFβ caused a statistically significant down-regulation of ALP mRNA in all co-cultures (*n*=4 per co-culture, Fig. 6C). Whereas ALP mRNA expression showed a large up-regulation after 28 days equally in all co-cultures, which is in line with the ongoing osteogenic differentiation (MSC_OB 25.92±7.29, OB_OB 53.10±15.98, MSC_MSC 30.56±10.35), addition of TGFβ caused a down-regulation of ALP in the MSC_OB co-culture (4.34±1.32, *p*=0.021), the OB_OB (15.50±4.06, *p*=0.021), and the MSC_MSC co-culture (5.59±1.96, *p*=0.021). The lowest ALP expressions in the groups without TGFβ treatment were found in the MSC_OB co-culture (25.93±7.30), followed by the MSC_MSC co-culture (30.57±10.34) and the OB_OB co-culture (53.10±15.99). In the groups with TGFβ there was no statistically significant difference between the MSC_OB and the MSC_MSC co-culture, but there was a statistically significant difference between MSC_OB and the OB_OB control (*p*=0.021).

Experiments investigating the mRNA expression of col1A1, Sp7, and ALP were repeated three times, also applying the different serum concentrations (2% and 5%), showing equal regulation patterns.

## Discussion

The most important finding of this study was the induction of calcification in MSCs by a co-culture with OBs, which was highly dependent on serum concentration. In contrast to standardized animal experiments, human donors demonstrated high individual variances in their differentiation potential, which were predictable based on the population doubling rate. Furthermore, the influence of TGFβ was remarkable leading to the induction of early markers of osteogenesis as collagen type 1 and Sp7 and inhibition of indicators of the final steps of osteogenic differentiation shown by suppression of ALP.

Tissue engineering of bone with progenitor cells, such as adult mesenchymal stem cells (MSCs), appears to be a promising strategy for the treatment of bone defects. The technique of co-culture is a possible strategy, which may avoid the application of specific differentiation stimuli. In fact, osteogenic differentiation of progenitor cells induced by co-culture with differentiated osteoblasts has been previously demonstrated by other groups.^6,7,12^ Whereas some publications emphasize the importance of cell-cell interactions,^7^ others demonstrated a paracrine mechanism^6^ as confirmed in the presented experiments. Although the principle of successful induction of an osteogenic phenotype in the progenitor cells by co-culture with OBs was previously described, for tissue engineering applications it is absolutely necessary to define culture conditions retaining the most efficient results. This is also important considering a possible generation of osteochondral constructs by MSC, in which paracrine effects of neighboring OBs and chondrocytes might initiate the differentiation of a chondral or osteogenic layer. Serum conditions were found to significantly affect proliferation and differentiation capacity of human adipose stem cells.^13^ Therefore, the effect of serum concentration was evaluated, showing the highest amount of calcium deposition when using a low serum environment.

Considering application in humans, a pronounced interindividual variation of differentiation capacity has to be taken into account. This could be confirmed by our experiments together with the opportunity to predict the differentiation potential by simply determining the population doubling rate of MSCs or OBs during the expansion period. An association of proliferation capacity with osteogenic differentiation potential in adipose derived MSC has been suggested before in rabbits, pigs and rats,^14^ but in the case of human donors may simply reflect biological cell health. During an amplification process osteoblasts or chondrocytes usually loose some characteristics of their original differentiation,^9^ but may restore the phenotype under cell-specific culture conditions. The presented correlation of proliferation with differentiation capacity does not indicate that the cells exhibit the osteogenic properties during the whole amplification period, it just refers to the capacity to restore the specific phenotype.

The role of TGFβ in osteochondral differentiation has been described, showing that proliferation of MSCs and their differentiation into chondrocytes is stimulated, osteoblast progenitor's differentiation into osteoblast is promoted, and hematopoietic stem cells are kept in a state of hibernation.^15^ It has been shown that high concentrations of TGFβ enhance OBs proliferation and down-regulates the expression of receptor activator of nuclear factor kappa B ligand, whereas low concentrations enhance osteoclast maturation.^15^ Similar to the presented data, Schagemann et al. have found that the initial release of TGFβ induced an expression of type 1 collagen and osteogenic marker genes in MSCs.^16^ Considering the constant and low dose TGFβ stimulus used in this experimental setup, it may be concluded that early markers of ossification, collagen type 1 and Sp7, were induced and indicators of the final steps of osteogenic differentiation such as ALP were inhibited. This is supported by a recently published clinical trial showing a correlation of serum TGFβ values with the incidence of non-unions in humans.^17^ A similar regulation pattern was observed in BMP-4 transfected C3H10T1/2 cells, in which the ALP peak values coincided with down-regulation of type 1 collagen.^18^ This is in line with the finding that Sp7 is necessary, but not sufficient for human MSC differentiation into osteoblasts.^19^

MSCs co-cultured with OBs showed levels of calcification measured by absorption at 450 nm and Alizarin red staining comparable to the positive controls of OBs alone or the co-culture of OBs and OBs. Whereas MSCs alone demonstrated only very low levels of spontaneous calcification, the addition of MSCs to MSCs in a co-culture resulted in a remarkable increase of osteogenic characteristics. Contrary to these results, mRNA levels in 1% FBS osteogenic medium did not show statistically significant differences in up- or down-regulation of the genes col1A1, Sp7, and ALP comparing the co-culture of MSCs and OBs with MSCs and MSCs. The discrepancy between calcification and gene expression might suggest that the OBs in the co-culture are mainly responsible for the calcification, possibly triggering by the secretion of soluble ALP. Since only the endpoint after 28 days has been analyzed, another explanation would be that ALP expression had already peaked earlier in the co-culture of MSCs with OBs as it has been described.^20^

Despite the fact that the data also add knowledge about natural repair mechanisms regarding osteogenesis, there are limitations caused by the study design. The experiments were all done in trans-well co-cultures using monolayers. The osteogenic differentiation cascade is of course influenced by a possible scaffold providing a three-dimensional environment,^21^ a factor, which was not addressed with the current experimental setup. Although the principal effects described could be observed independent of the donors, high interindividual discrepancies were observed. When interpreting the data, the limited number of cell donors also needs to be kept in mind. This issue is always relevant when working with human specimens, but may be addressed by the estimation of differentiation potential using the population doubling rate. Furthermore, the experimental design only allows the paracrine effects to be evaluated and neglects cell–cell interactions.

To summarize, the data of this study indicate that low serum conditions support osteogenesis and that TGFβ mediates the initial molecular steps but counteracts final differentiation. The interindividual variations in humans are high, but may be predicted to a certain degree by observing cell functions a proliferation capacity.

## Abbreviations Used

ALP: alkaline phosphatase
ARS: Alizarin red staining
col1A1: collagen type 1
DMEM: Dulbecco's modified Eagle's medium
FBS: fetal bovine serum
MSC: mesenchymal stem cells
OB: human osteoblasts
OD: optical density
PDR: population doubling rate
TGFβ: transforming growth factor beta
